# High Tibial Valgus Osteotomy: Closing, Opening or Combined? Patellar Height as a Determining Factor

**DOI:** 10.1007/s11999-014-3821-5

**Published:** 2014-07-29

**Authors:** Oliver Portner

**Affiliations:** Division of Orthopaedic Surgery, University of Ottawa, 75 Pond Street, Ottawa ON, K1L 8J1 Canada

## Abstract

**Background:**

According to the literature, closing and opening wedge high tibial valgus osteotomies can raise or lower the patella, and diffèrent methods of patella height measurement show similarly conflicting results. Clarification of this was thought to be important because there is much literature describing morbidity secondary to patella alta or patella infera (baja). Effects on tibial slope and patellar tendon length are not well delineated and the influence of sex and age is unknown.

**Questions/purpose:**

A group of patients who underwent high tibial valgus osteotomy was investigated to determine how surgical technique influenced postoperative (1) patellar height and (2) tibial slope and patellar tendon length, and (3) whether age or gender independently influenced postoperative patellar height. To eliminate the often conflicting results seen when several ratio methods are used, patellar height was measured by one method, before and after surgery, shown previously to be reliable.

**Methods:**

Patellar height was measured on radiographs using the plateau-patella angle in a retrospective case series consisting of three cohorts: 18 patients with closing wedge osteotomies, 26 with opening wedge osteotomies, and 32 with combined osteotomies. The indication for surgery in all three cohorts was medial osteoarthritis with secondary varus. Before surgery there were no significant differences in patellar height, femorotibial angle, age, or gender among the three groups, and no patients were lost to followup during the 8-week study period after surgery. Seven of the 76 patients (9.2%), all in the opening wedge cohort, had concomitant ACL reconstruction at the time of the tibial osteotomy. No other surgery, except arthroscopy, was performed at the time of osteotomy. Patellar tendon length was assessed by the Insall-Salvati index and tibial slope by the angle between the posterior tibial cortex and the medial tibial joint line. Postoperative measurements were made between 6 and 8 weeks. The influence of sex and age was calculated using patellar height measurements made before any surgery.

**Results:**

All closing wedge osteotomies produced patellar ascent by an average of 13% (p < 0.001), all opening wedges produced descent by an average of 21% (p < 0.001), and the combined osteotomy mean showed minimal change (p = 0.0034). The absolute consistency of the changes and their direction allow suggested guidelines for selection of osteotomy type. There were only slight changes in tibial slope. A significant change in patellar tendon length was seen in seven knees of the opening wedge cohort that had concomitant ACL reconstruction. All had a mean reduction of the Insall-Salvati index of 0.05 (approximately 5%), p = 0.0002. New findings showed higher patellae in female and older patients, unrelated to the surgery.

**Conclusions:**

If it is accepted that patella baja and patella alta should be avoided, then closing wedge osteotomies should be performed only when the patella is low riding, and opening wedge osteotomies should be done only for patients with preexisting patella alta. The combined osteotomy minimizes changes in patellar height. Patellar tendon contractures and tibial slope changes can be avoided. The plateau-patella angle should be measured preoperatively to help decide the type of osteotomy.

**Level of Evidence:**

Level III, therapeutic study. See the Instructions for Authors for a complete description of levels of evidence.

## Introduction

High tibial valgus osteotomy, popularized by Coventry [9], is an important treatment option to unload the painful degenerative medial compartment in younger adults, but it is known to affect patellar height. Patellar height variations have been implicated in causing patellar dislocations, patellofemoral arthrosis (especially in patella infera), and technical problems relating to knee arthroplasties [1–5, 10, 19, 20, 29, 30].

Theoretically, in supratubercle osteotomies, a closing wedge should mechanically raise the patella by lowering the joint line, opening wedge osteotomies should cause descent by distalizing the tubercle, and a combination of lateral closing and medial opening wedges (combined osteotomy), should produce a patellar height closer to neutral. Patellar tendon contractures, presumably produced by the local trauma of surgery, should lower the patella in any type of high tibial osteotomy, and likely would be exacerbated by immobilization.

However, there is confusion regarding the effect of a high tibial osteotomy on patellar height for closing and opening wedge osteotomies [18, 27, 28]. Elevation and lowering of the patella have been reported for both techniques, often opposite to mechanical expectations [18, 27, 28]. There has been more recent agreement that the opening wedge osteotomy may lower the patella [10, 27, 31]. The inconsistent results may be attributable to the various methods commonly used to measure patellar height, to patellar tendon contractures, and to differing postoperative rehabilitation regimens. The contractures have been blamed for patellar lowering in any type of osteotomy [7, 8], but it seemed likely that early motion might prevent them. Another associated issue has been the effect on tibial slope in which an increase has been associated with opening wedge osteotomies [21, 24, 31], and a decrease with closing wedge osteotomies [11, 25, 31]. The current study was designed to see whether eliminating some of the perceived causes of the uncertainty would result in confirmation that closing wedge osteotomies should consistently raise the patella, that opening wedge osteotomies should lower it, and that combining a lateral closing wedge with a medial opening wedge could result in less raising or lowering.

A group of patients who underwent three different types of high tibial valgus osteotomy was investigated to determine how surgical technique influenced (1) postoperative patellar height, (2) tibial slope and patellar tendon length, and (3) whether gender or age independently (ie, unrelated to surgery) influences patellar height.

## Patients and Methods

A retrospective case series was evaluated, on radiographs only, consisting of 18 closing wedge osteotomies, 26 opening wedge osteotomies, and 32 combined osteotomies, for a total of 76 osteotomies. One surgeon (OP) performed the closing wedge and combined osteotomies, and another (GD) performed the opening wedge osteotomies. There was no preselection of patients other than to include patients who had only supratubercle osteotomies, those with sufficient flexion on the radiographs (30° or greater) to tighten the extensor mechanism, and those with available appropriate preoperative and postoperative digital radiographs. Quadriceps bulk, strength, and degree of relaxation at the time of radiography were not evaluated. The indication for surgery in all patients was painful medial osteoarthritis with relative varus in patients who were deemed unsuitable for a unicompartmental arthroplasty because of young age or because the degree of osteoarthritis was not thought to be sufficiently severe. No cases were discarded unless the radiographs were excluded, and no patients were lost to followup during the 8-week study period after surgery. Preoperative patellar height was comparable in the three cohorts. The closing wedge osteotomies were done between February 13, 2007 and November 16, 2010, the opening wedges between December 12, 2006 and January 10, 2012, and the combined osteotomies between September 6, 2009 and February 10, 2012. In a one-way ANOVA study, these cohort numbers and their differences yielded 100% power.

Knees with excessive rotation observed on radiographs (three), or with abnormal bony anatomy from previous surgery (three), other than ACL reconstruction tunnels, were the only exclusions.

Demographics recorded included age, which ranged from 21 to 59 years with a mean of 46 years; side of surgery, the right side predominating in all three cohorts; sex, with males representing 79% of the total; and a history of previous surgery (other than simple arthroscopy) in only seven patients, all of whom were in the opening wedge group (Table 1). These seven patients had undergone six ACL reconstructions and one distal patellar realignment (Elmslie-Trillat) before osteotomy. In addition, at the time of the osteotomy, an additional seven patients, also in the opening wedge group only, had concomitant ACL reconstructions, four of which were revisions.

**Table 1:** Baseline demographics

Demographic	Closing wedge osteotomy (n = 18)	Opening wedge osteotomy (n = 26)	Combined osteotomy (n = 32)	Total (n = 76)	p value*
Age	46.5 years (5.17)	43.9 years (8.48)	47.5 years (5.97)	46.0 ± 6.87 years	0.134
Mean ± SD (range)	(36–54 years)	(21–59 years)	(29–56 years)	(21–59 years)	
Sex					
Female	3 (16.7)	6 (23.1)	7 (21.9)	16 (21)	0.867
Male	15 (83.3)	20 (76.9)	25 (87.1)	60 (79)	
Side					
Left	6 (33.3)	11 (42.3)	15 (46.9)	32 (42.1)	0.648
Right	12 (66.7)	15 (57.7)	17 (53.1)	44 (57.9)	
Previous surgery** Number	0	7 (27%)	0	7 (9.2%)	

* P values are from one-way ANOVA for continuous variables and from chi-square test for categorical variables; **excluding arthroscopy.

**Fig. 1:**
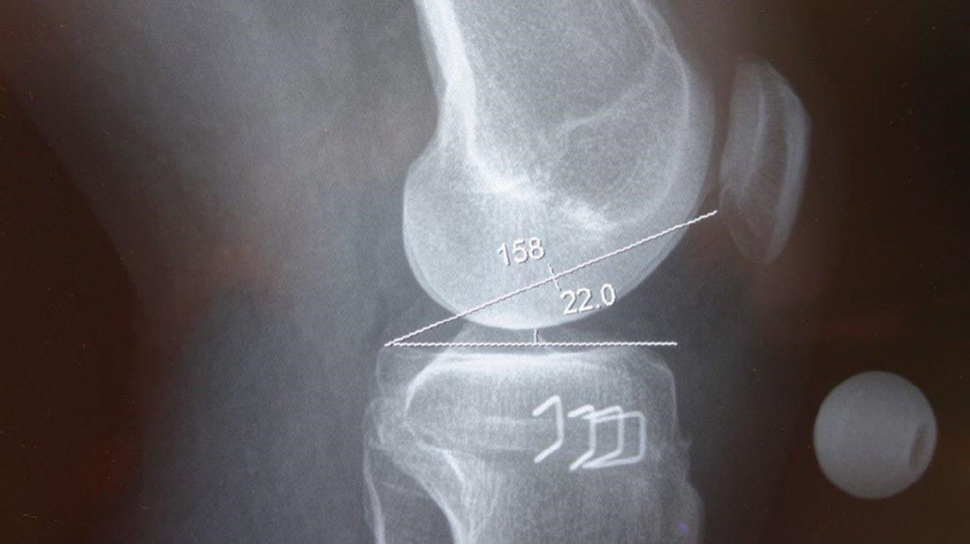
The postoperative plateau-patella angle measured 22° on the radiograph. Before surgery, it was 21.6°.

**Fig. 2A–B:**
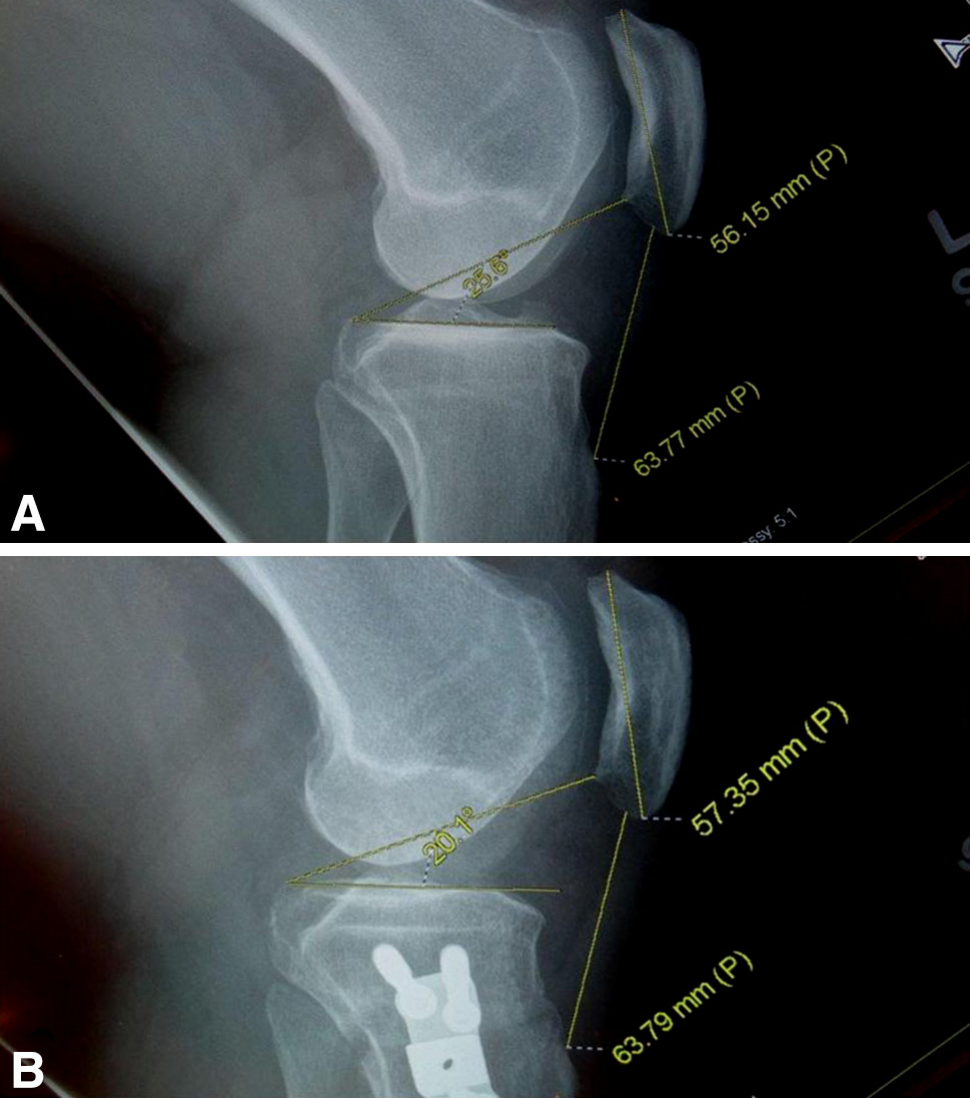
(**A**) Before the opening wedge osteotomy, the plateau-patella angle measured 25.6° and the Insall-Salvati index was 1.14 (63.77 ÷ 56.1). (**B**) After the osteotomy, the plateau-patella angle measured 20.1° and the Insall-Salvati index was 1.11 (63.79 ÷ 57.35).

**Fig. 3:**
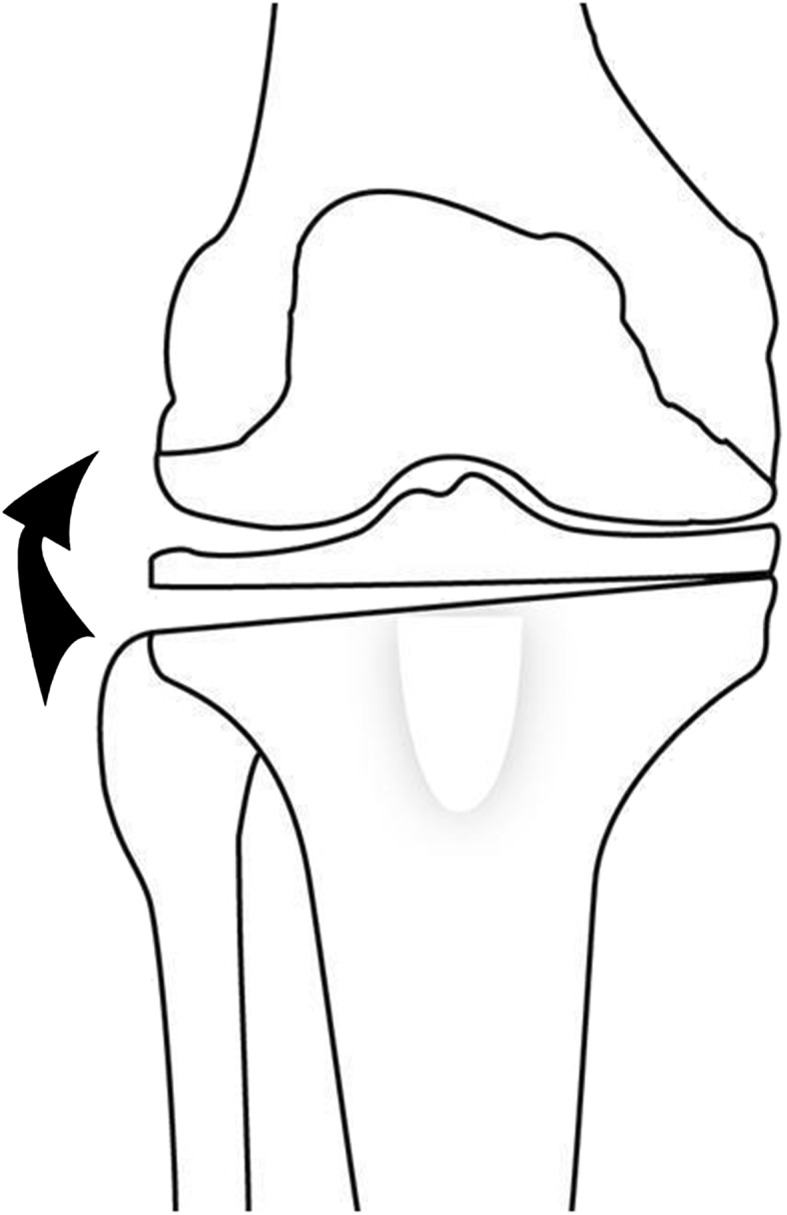
The drawing shows the lateral closing wedge osteotomy, with wedge removed.

**Fig. 4:**
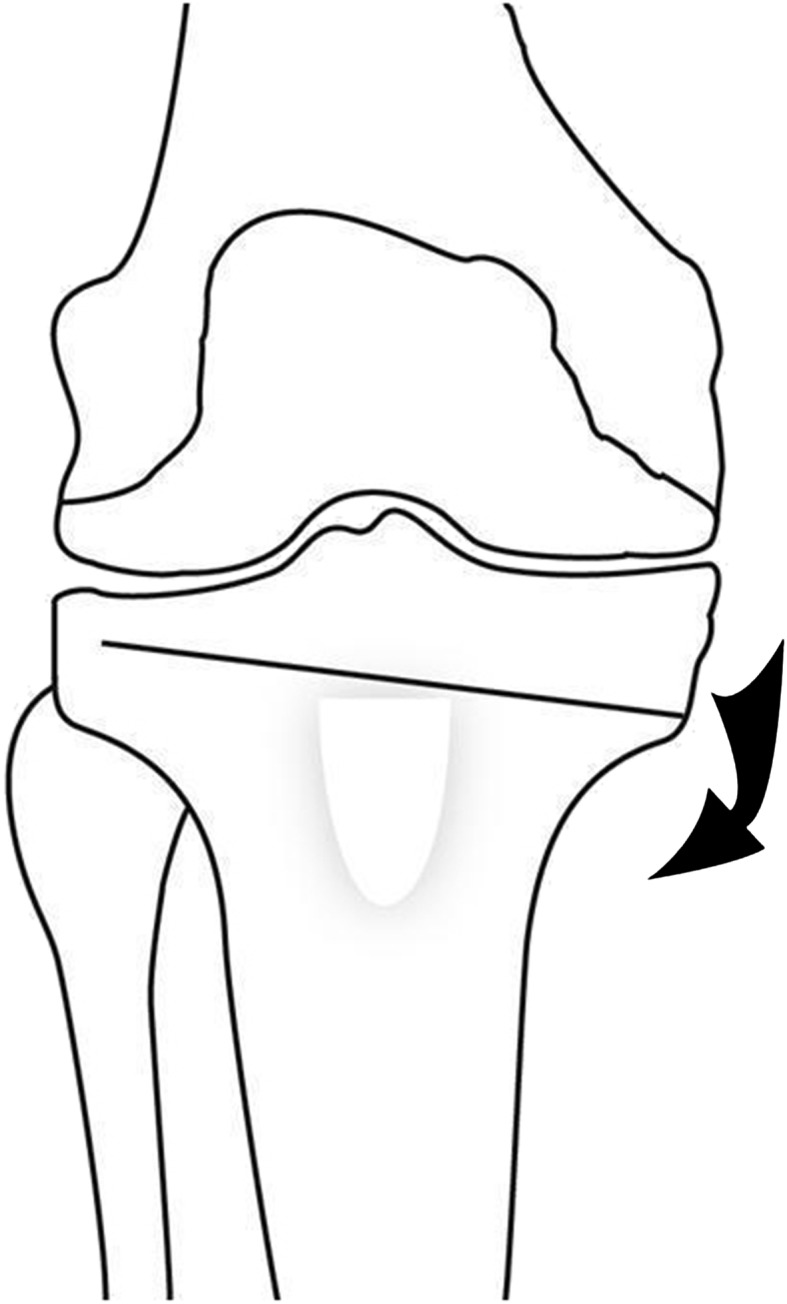
The medial opening wedge osteotomy before opening is shown in this drawing.

AP and lateral digital radiographs of the knees were assessed by the author with all measurements made using built-in software (Agfa IMPAX 6.5, Waterloo, Ontario, Canada) for the closing and combined osteotomies, and McKesson version 12 (San Francisco, CA, USA) for the opening wedge osteotomies. This was done preoperatively, and in all patients between 6 and 8 weeks postoperatively. There was no change between these measurements and those taken at 3 months, but these data were incomplete and therefore not included. Long-term changes on the basis of progression of arthritis and other factors are possible. No patients were lost to followup before 6 months and all were clinically and radiographically assessed by their surgeon before this time. Four parameters were measured: (1) femorotibial angle, to record changes in the anatomic axis, using lines drawn through the center of the medullary canals and done for completeness and to ensure the cohorts were comparable; (2) plateau-patella angle to measure patellar height [26], using the angle between a line tangential to the medial tibial plateau and the line from the posterior extent of the latter to the inferior articular margin of the patella on the lateral view (Fig. 1); (3) the Insall-Salvati index, the ratio between the length of the deep surface of the patellar tendon and the diagonal length of the patella [16], to evaluate possible patellar tendon contractures (Fig. 2), and (4) tibial slope was measured using the angle between the joint line (medial tibial plateau) and the posterior tibial cortex as described by Brazier et al. [6].

The study by Portner (the current author, and who devised the method) and Pakzad showed excellent interobserver and intraobserver reliability of the method and simplicity of use compared with other commonly used methods of patellar height measurement. To evaluate the influence of sex and age, the plateau-patella angles from the previously studied cohort of 269 knees (unrelated to surgery) [26] were combined with the preoperative measurements of the 76 in the current study to increase power. The data then were analyzed statistically. This was not done in the previous study which used data only in the aggregate and did not look at these two variables. The previous study was done specifically to describe and validate the plateau-patella angle and to compare it with the three most commonly used methods of measuring patellar height (Insall-Salvati, Blackburne-Peel, and Caton-Deschamps). Measuring the plateau-patella angle in those 269 knees yielded a mean patellar height of just less than 25°, and one standard deviation above and below this was used to define patella alta at greater than 29° and patella infera at less than 21°.

In addition, for 20 patients who had combined osteotomies, an attempt was made to identify, on postoperative radiographs, the pivot point between the closing wedge and opening wedge to assess the degree to which its location might predict changes in patellar height.

### Surgical Techniques

For the closing wedge osteomy, a laterally based wedge (Fig. 3) was cut approximately 1½ cm below the joint line and above the tibial tubercle, sloping somewhat distally toward the medial side and leaving a small medial cortical hinge to maintain stability. No fibular osteotomies (other than occasional rongeuring of a small amount of fibular head in larger corrections) or ligament releases were performed. The osteotomy was closed, lateral fixation was done using a staple gun, and a bulky compression bandage was applied from the groin to the ankle and left in place overnight.

An opening wedge osteotomy (Fig. 4) was performed in a standard manner above the tibial tubercle. The opening amount was adjusted according to the hip-knee-ankle axis, evaluated by intraoperative fluoroscopy. Care was taken to avoid greater distraction anteriorly to prevent increasing the sagittal tibial slope. Fixation was done using a plate and screws.

**Fig. 5:**
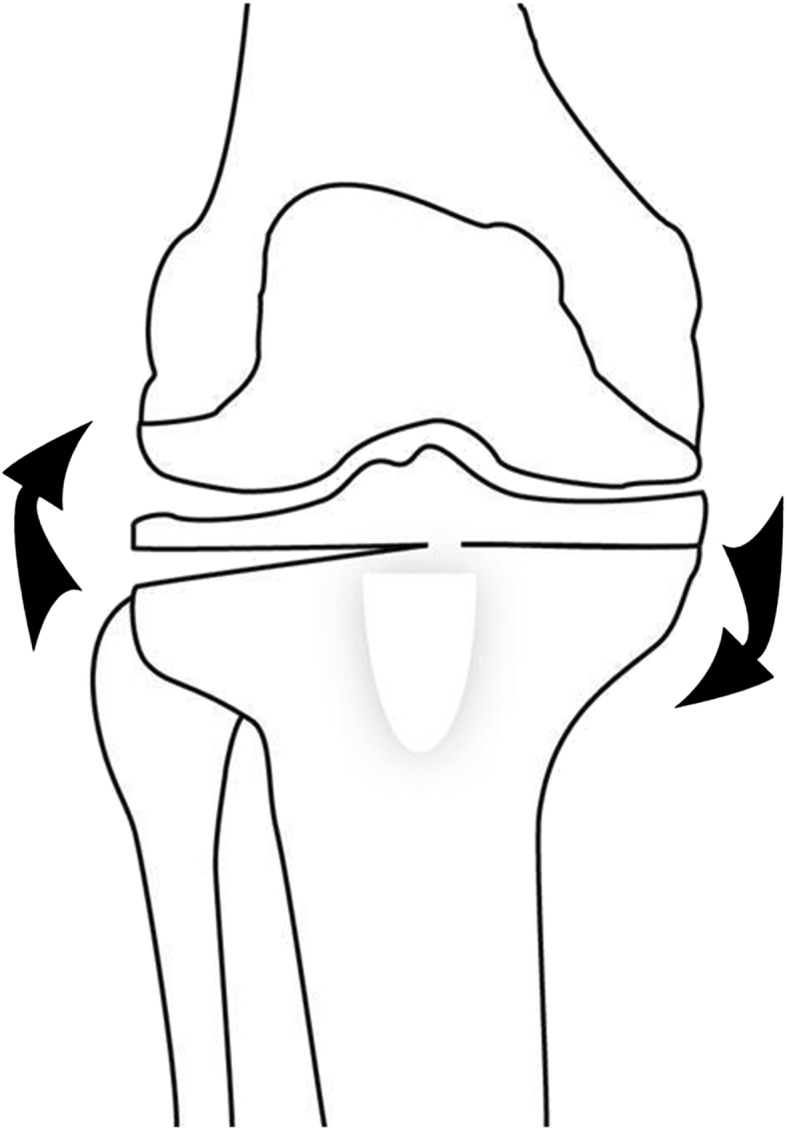
The drawing shows the combined lateral closing osteotomy with the wedge removed, with a medial opening wedge osteotomy.

In the combined lateral closing with medial opening wedge osteotomy (Fig. 5), a laterally based wedge was removed, but with the apex beneath the medial margin of the patellar tendon instead of at the medial cortex. A straight medial osteotomy then was made from under the medial collateral ligament to within 2 to 3 mm of the apex of the lateral wedge. This resulted in a small bridge of intervening bone to provide a hinge for stability and to avoid tibial slope change. The wedge of bone was removed, reversed, and inserted medially under the intact medial collateral ligament. Staples were placed laterally, and closure and bandaging were done as for the closing wedge osteotomies.

All patients began ROM exercises within 24 hours of surgery. A knee immobilizer was worn for 6 weeks and was removed only for frequent flexion and extension exercises and bathing. Resistance exercises were started in the sixth week along with low-tension stationary cycling. Partial weightbearing progressing to full weightbearing as tolerated was allowed immediately for patients who had closing wedge osteotomies, at 3 to 4 weeks for patients who had combined osteotomies, and at 8 weeks for patients who had opening wedge osteotomies.

### Statistical Methods

Demographics of age, sex, and side of surgery were compared for each of the three patient cohorts using ANOVA for continuous variables and chi-square for categorical variables. Measurements of femorotibial angle, tibial slope, plateau-patella angle, and Insall-Salvati ratios were charted for each of the 76 knees, before and 6 to 8 weeks after surgery. The means, SDs, ranges and differences, comparing the preoperative and postoperative values in the three cohorts, using ANOVA for group comparisons and paired t-tests in each group are shown (Table 2). The two-sample t-test was used to compare the plateau-patella angle in males and females (Table 3), and the Pearson correlation coefficient to assess correlation between plateau-patella angle and age.

**Table 2:** Measured characteristics of the three cohorts

Variable	Closing wedge osteotomy (n = 18)	Opening wedge osteotomy (n = 26)	Combined osteotomy (n = 32)	p value*
Femorotibial angle [Mean ± SD (range)]				
Preoperative	1.59 ± 1.37 (−1.1 to 3.4)	−0.28 ± 2.99 (−5 to 4.5)	0.21 ± 2.38 (−7 to 3.6)	0.0589
Postoperative	7.57 ± 2.32 (1.2–12.5)	7.48 ± 2.34 (2.8–12)	8.07 ± 2.14 (3.8–12.4)	0.563
Change for postoperative from preoperative	5.87 ± 2.12 (1.6–10.2)	7.76 ± 3.57 (1.6–14)	7.78 ± 2.45 (4.4–13.1)	0.0579
p value**	< 0.0001	< 0.0001	< 0.0001	
Patellar height [Mean ± SD (range)]				
Preoperative	24.28 ± 3.38 (17.3–28.9)	22.83 ± 2.95 (17.2–28.3)	23.8 ± 3.01 (18.8–32.2)	0.278
Postoperative	27.37 ± 4.02 (18.2–34)	18.15 ± 3.20 (12.3–24.5)	24.97 ± 2.85(20.3–32.7)	< 0.0001
Change for postoperative from preoperative	3.1 ± 2.29 (0.7–7.6)	−4.70 ± 2.27 (−8.6 to −0.9)	1.17 ± 2.08 (−2.5 to 7.9)	< 0.0001
p value**	< 0.0001	< 0.0001	0.0034	
Tibial slope [Mean ± SD (range)]				
Preoperative	3.32 ± 3.15 (−3.4 to 8.5)	5 ± 3.68 (−0.7 to 14.2)	5.75 ± 2.87 (0.8 to 11.4)	0.0442
Postoperative	2.27 ± 3.78 (−6.4 to 8.7)	5.43 ± 4.32 (−2.1 to 14.7)	4.96 ± 2.66 (−1 to 10.4)	0.0125
Change for postoperative from preoperative	−1.05 ± 2.45 (−7 to 2.1)	0.43 ± 2.6 (−4.2 to 7.3)	−0.78 ± 1.66 (−4.4 to 2)	0.0517
p value**	0.086	0.398	0.012	
Insall-Salvati index [Mean ± SD (range)]				
Preoperative	0.96 ± 0.19 (0.65–1.33)	0.96 ± 0.13 (0.72–1.14)	0.98 ± 0.15 (0.69–1.4)	0.933
Postoperative	0.98 ± 0.22 (0.66–1.44)	0.97 ± 0.13 (0.69–1.15)	0.98 ± 0.15 (0.67–1.14)	0.930
Change for postoperative from preoperative	0.02 ± 0.06 (−0.06 to 0.15)	0.0004 ± 0.039 (0.07–0.08)	0.005 ± 0.03 (−0.09 to 0.08)	0.442
p value**	0.233	−.960	0.404	

* P values for comparing the three treatment groups at the same times are from ANOVA; **p values comparing scores with time in the same treatment group are from paired analysis.

**Table 3:** Differences in plateau-patella angle between sexes

Parameter	Males	Females	Two-sample t-test p value
Patellar height mean (SD)	24.17 (3.25)	25.31 (3.68)	0.0028

## Results

**Fig. 6:**
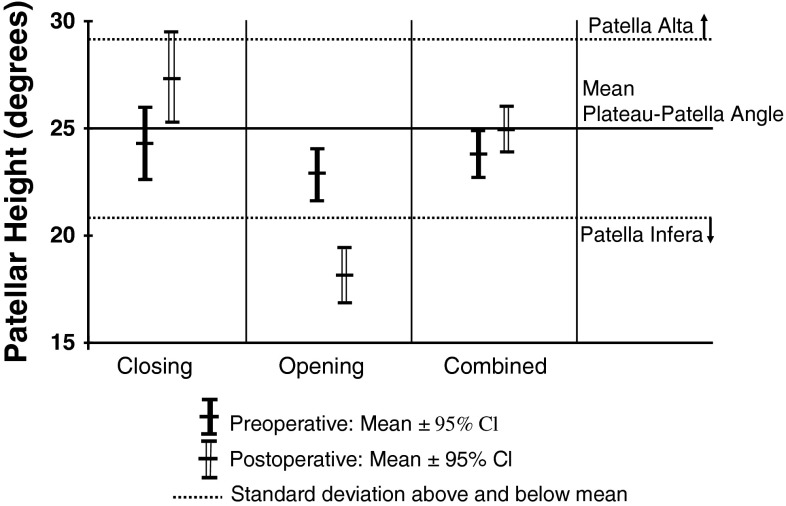
The plateau-patella angles before and after surgery in the three cohorts with 95% CI are shown.

**Fig. 7:**
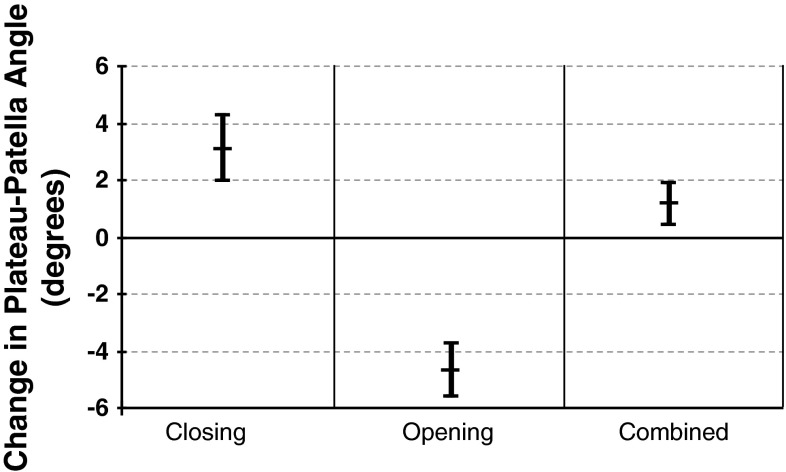
The mean change and 95% CI for the plateau-patella angle in each the three cohorts are shown.

Patellar height measurements confirmed the theoretically expected effects of high tibial osteotomy on patellar height (Table 2). The changes are shown for angle change (Fig. 6) and for mean changes (Fig. 7), illustrating the extent of the changes in each type of osteotomy. One hundred percent of closing wedge osteotomies raised the patella by a mean of 3.1° (SD 2.3°; p < 0.001), or 13% of average preoperative patellar height, resulting in six knees (19%) with patella alta. There were two preoperative cases of patella infera which were improved but not converted to patella norma.

One hundred percent of opening wedge osteotomies resulted in descent of the patella by a mean of 4.7° (SD, 2.3°; p < 0.001), or 21% of average preoperative patellar height. After surgery, 22 of 26 knees (85%) had patella infera, although seven (27%) had patella infera even before surgery. No patella alta was seen in this cohort before or after surgery. In the patients who had combined osteotomy, the patella was either raised (22) or lowered (10), but by smaller amounts. The resulting mean was a raise of 1.2° (SD, 2.1°; p = 0.003), or approximately 5% of average preoperative patellar height. In 20 knees the width of the closing component was estimated to be approximately 55% of the width of the tibia. Although not accurate, it did suggest that there was more closing wedge than opening, which would suggest some elevation of the patella.

Tibial slope measurements showed only small changes in all three cohorts, with means of −1.1° in the closing wedge cohort (p = 0.086), +0.4° in the opening wedge cohort (p = 0.398), and −0.8° (p = 0.012) in the combined cohort. The frequently reported marked increase of tibial slope in opening wedge osteotomies [21, 24, 25, 31], and the opposite in closing wedge osteotomies [8, 9, 24, 25], generally were avoided. With respect to patellar tendon contractures, means of the Insall-Salvati index showed no differences with the numbers available (69 knees). In a subset of seven patients in the opening wedge cohort with concomitant ACL reconstruction, however, a mild patellar tendon contracture resulted, with a mean Insall-Salvati index change of −0.05 (5%), compared with +0.01 in the remaining 19 patients in this cohort (p < 0.001), of whom none had additional surgery, other than arthroscopy.

In the analysis of sex and age, the mean plateau-patella angle was 24.2° ± 3.3° for male patients (n = 216), and 25.3° ± 3.7° for female patients (n = 129). Thus the female patients had a significantly higher patellar height than males (p = 0.0028), averaging 5% more. Older age was associated with increasing patellar height with the Pearson correlation coefficient showing a statistically significant, although weak, correlation of 0.14 (p = 0.0096).

## Discussion

Numerous studies associate patella alta and infera with dislocation, chondromalacia, osteoarthritis, and technical difficulties relating to knee arthroplasty [1–5, 10, 14, 19, 20, 29, 30]. Other studies of high tibial osteotomy result in confusion regarding whether the patella is raised or lowered, and the effects of these osteotomies on tibial slope and patellar tendon length are not clear. Although the reasons for the discrepancies are not known, it was suspected that the array (two or three usually in each study) of different methods used to measure patellar height give conflicting results. The plateau-patella angle was designed [26] to yield an absolute number (degrees) rather than a ratio, and for consistency, was the only method used here. The study was performed to elucidate the confounding factors and show predictability in the effect of high tibial osteotomy on patellar height. The expected ascent of the patella in closing wedge osteotomies and descent in opening wedges was confirmed with no exceptions, tibial slope alteration was thought to be controllable but occurs, and patellar tendon contractures were found only with concomitant surgery of some magnitude. Ideally, any surgery on the knee should not significantly worsen the position of the patella, making the combined osteotomy an option worth considering to avoid patellofemoral issues.

One of the limitations of the study is the reliance on existing literature associating higher or lower patellae with knee problems. Experience shows that not all high- or low-riding patellae are problematic, but when actual patella alta or infera is diagnosed (ie, above or below a certain limit, defined here as one standard deviation), concern should be raised. A second is that so far, no single method of measuring patellar height has been accepted as the gold standard, which is probably why numerous previous studies have used two or three methods. Each method has its own particular limitations, including that small differences from measurement error occur in all. In the plateau-patella angle, measurement error is in the range of 1° or less, while it is somewhat more difficult to discern what it might be in the ratio methods. A third limitation is that this study focuses only on the first 8 weeks after surgery, since it is the author’s experience that measurements do not change after this period of bone healing and appropriate rehabilitation, except possibly in the much longer term if the degenerative arthritis progresses. The clinical followup of the patients was not in the scope of this study. Assessor bias is possible, since all measurements were performed by the author alone, relying on a previous study [26] which showed good interobserver and intraobserver reliability of the method.

Studies published on patellar height after high tibial osteotomy usually use two or three of the Insall-Salvati, Blackburne-Peel, and Caton-Deschamps indices for its measurement, often with confusing results [18, 27, 28]. Explanations for the discrepancies were not reported, except for the series in which postoperative plaster casts were used in closing wedge osteotomies [28], likely resulting in scarring down or contractures of the patellar tendon. Thus tendon contractures are one possible explanation, leaving measurement uncertainty as another. The current study deals with the latter by using only one method of measurement, and tracks down only one cause of contractures in the former to concomitant surgery. Of the common measurements of patellar height, the least consistent is the Insall-Salvati index, which is difficult to measure accurately [26], except sequentially in the same knee, since the four measuring points are unique and will not change in the same knee, unless deformed by the surgery. Thus, it is most valuable for specific evaluation of the length of the patellar tendon, especially in conjunction with knee surgery. Given its variability, it is less good as a general assessor of patellar height. The plateau-patella angle is measured easily without calculations, giving a good range from patella infera to patella norma to patella alta [26]. It consistently measured increased values in closing wedges and decreased values in opening wedges.

Patellar height change also can be avoided in high tibial osteotomies by leaving the tibial tubercle attached to the proximal segment [12, 15, 17, 22], or by detaching and then reattaching the tubercle after the osteotomy [13]. These techniques are technically demanding and need solid fixation to allow the early ROM necessary to avoid patellar tendon contractures [8].

Tibial slope changes were evaluated because of studies that associate an increased slope with opening wedge osteotomies [21, 24, 25, 31], and closing wedge osteotomies with a decreased slope [7, 11, 25, 32]. The minimal mean change in tibial slope in all three cohorts in this study shows that it can be largely controlled by careful attention to surgical technique, but this did not happen in every case. The decreased slope associated with closing wedge osteotomies is likely related to the greater difficulty and risk of removing posterior bone which then results in a posterior gap that is smaller than the anterior gap [32]. In opening wedge osteotomies, however, the increased slope arises because the surgical approach and biomechanics make it difficult to maintain the posterior gap open as much as the anterior gap. LaPrade et al. [21] found that tibial slope increased by an average of 4.3° when the plate was positioned anteromedially compared with the smaller change of 1° when placed posteromedially. Chae et al. [8] described avoiding change in slope by using bone graft and fixation such that the ratio of anterior to posterior gap was approximately 2:3.

No references to the influence of sex and age were found in the literature, and the causes of the findings here, of higher patellae in women and older patients, are unknown. It could be speculated that they may be attributable to hormonal differences, along with aging collagen.

The combined osteotomy offers some advantages over traditional types: the removed wedge is thinner (for the same degree of correction), but still can tighten the medial collateral ligament and, in closing wedges, decrease the slackness produced in the lateral collateral ligament, it allows the ability to adjust the patellar height as desired, and it rarely needs bone grafting. Two other, fairly similar techniques of combined osteotomy have been reported [23, 25].

Based on this study, it is recommended that (1) surgeons avoid lateral closing wedge osteotomies when the patella is already very high (plateau-patella angle greater than 29°), and (2) that they avoid opening wedge osteotomies when the patella is lower than the normal mean of 25° (½ the population). The combined osteotomy confers the ability to minimize patellar height change, or to correct abnormal patellar height, by adjusting the proportions of the closing and opening wedges. Tibial slope changes and patellar tendon contractures can be largely avoided by assiduous attention to surgical technique with the former and early ROM exercises with the latter. The Insall-Salvati index is useful to show changes in tendon length in conjunction with another method to show changes of patellar height produced by repositioning the tibial plateau. The addition of concomitant surgery, such as ACL reconstruction, may contribute to patellar tendon contracture, and thus patellar lowering. Females have higher-riding patellae than males, and advancing age appears to increase patellar height. Patellar height should be measured preoperatively to help determine the method of osteotomy, thus avoiding a change in height which could cause future problems. The plateau-patella angle is a simple measurement that allows the necessary quantification.
